# Editorial

**DOI:** 10.1093/europace/euae008

**Published:** 2024-01-11

**Authors:** Angelo Auricchio

**Affiliations:** Istituto Cardiocentro Ticino - Ente Ospedaliero Cantonale, Via Tesserete 48, 6900 Lugano, Switzerland

It is now a year since the new Editorial Board of *Europace*—EHJ Arrhythmias and Electrophysiology has been installed. The year 2023 marked an important point of transition for Journal activities not limited to the change in the members of the Editorial Board.

The transition to a fully open access publishing model has possibly represented the most remarkable change. From January 2023, all papers published in *Europace* have been freely available to all readers. That means the research in the Journal had a wider reach and can therefore be expected to have a wider impact. We are aware that this major change in publication mode has been criticized by some authors. The unchanged number of submissions (with a growing trend during the most recent months), however, shows that the criticism to open access is unjustified as long as quality in the peer review process, efficient manuscript handling, fast production, and high dissemination of Journal content are satisfying authors’ expectations. Many more colleagues worldwide—especially those practising in less wealthy geographies—immensely benefited by the transition to an open access. We also emphasize the significant publication discount that European Heart Rhythm Association (EHRA) members may enjoy as well as the fee waiver agreement for papers where a corresponding author submits from a country listed in Oxford University Press (OUP)’s developing countries initiatives (https://academic.oup.com/pages/purchasing/developing-countries-initiative/participating-countries-and-regions). Additionally, OUP has a large number of ‘Read & Publish’ agreements in place, which means many authors will have their open access fees covered by their institution. To learn if your institution has a Read & Publish agreement in place and to find out how to check whether your paper can be covered by the funding available, please see https://academic.oup.com/pages/open-research/read-and-publish-agreements/participating-journals-and-institutions.

The year 2023 represented an important landing and starting point of *Europace* activity. The Journal commemorated its 25th year of continuous publication^1–12^ and the 20 years of the activities of the EHRA.^13^ To honour these notable achievements with the deserved emphasis, a Journal issue has been dedicated to the EHRA 20-year anniversary and two others to the 25th Journal anniversary.

The Journal’s April issue contains a historical perspective of EHRA’s foundation and its development, presenting the reached milestones.^1^ The EHRA was created on 2 September 2003 with a mission of ‘improving the quality of life and reducing sudden cardiac death by limiting the impact of heart rhythm disturbances’. Over the years, the EHRA has become the leading European network and one of the most influential organizations worldwide for heart rhythm specialists and allied professionals.

As far as the celebration of Journal activities during the last 25 years is concerned, the Journal Editorial Board commissioned a large group of scientists to write a series of state-of-the-art reviews. These reviews provide an historical overview and key scientific contributions of the Journal to the world community of cardiac electrophysiologists, cardiologists, and those physicians interested in the diagnosis, management, and treatment of various arrhythmia disorders. The state-of-the-art reviews appeared in the August and September issues of *Europace*—EHJ Arrhythmia and Electrophysiology.

In 2023, the Journal’s Impact Factor reached 6.1, thus being the third in the specialty field of arrhythmias and electrophysiology and the 29th out of 210 journals in the field of cardiac and cardiovascular systems. The Journal editors express their profound gratitude and appreciation to those authors who have submitted to the Journal and will continue support its further development by publishing high-quality research and novel, clinically relevant study results. Notably, in 2023, the Journal had the opportunity to publish several manuscripts accepted as late breaking trials at different international scientific meetings. We now encourage authors to submit by 24 February 2024 their petition for fast-track review status for a late breaking trial submitted to the EHRA 2024 Congress. The author will be notified within 48 h whether the manuscript is approved for fast-track review. Once approved, the initial decision will be made within 5 days. If provisionally accepted, a revised manuscript must be returned to the Editorial Office as stipulated in the decision letter. If the manuscript is accepted for publication, it will be embargoed until the official date of presentation at the EHRA 2024 meeting. The open access publishing fee will be waived for the top five rated accepted trials.

I am very much looking forward to another productive, engaging, and enjoyable year conducive to high-quality scientific and educational Journal content. This is also the right time to give my heartfelt thanks to all of the members of the *Europace* Editorial Board for their time, passion, and support of the Journal. A special thanks goes to the Executive, Deputy, and Associate Editors. They have immensely contributed to the Journal’s success, not only as handling editors or manuscript reviewers but also as advisers at the time of challenging decisions, coaches to junior editors, and as promoters by disseminating via social media Journal content. My profound gratitude is for all Editorial Consultants who spend time to critically review many manuscripts and to provide invaluable feedback to manuscript authors; without their restless activity, our job would be impossible! Finally, I like to take this opportunity to thank everyone at OUP for their unrestricted commitment to the new Editorial Board and all of you who in the past have continuously sustained the Journal as authors and readers and will continue to do so in the future!

Best personal regards,

Angelo Auricchio
